# Effects of Mental Workload Manipulation on Electroencephalography Spectrum Oscillation and Microstates in Multitasking Environments

**DOI:** 10.1002/brb3.70216

**Published:** 2025-01-08

**Authors:** Wenbin Li, Shan Cheng, Jing Dai, Yaoming Chang

**Affiliations:** ^1^ Department of Aerospace Hygiene, Faculty of Aerospace Medicine Air Force Medical University Xi'an China; ^2^ Department of Aerospace Medical Equipment, Faculty of Aerospace Medicine Air Force Medical University Xi'an China; ^3^ Department of Aerospace Ergonomics, Faculty of Aerospace Medicine Air Force Medical University Xi'an China

**Keywords:** band power, EEG microstate, mental workload, multitasking

## Abstract

**Introduction:**

Multitasking during flights leads to a high mental workload, which is detrimental for maintaining task performance. Electroencephalography (EEG) power spectral analysis based on frequency‐band oscillations and microstate analysis based on global brain network activation can be used to evaluate mental workload. This study explored the effects of a high mental workload during simulated flight multitasking on EEG frequency‐band power and microstate parameters.

**Methods:**

Thirty‐six participants performed multitasking with low and high mental workloads after 4 consecutive days of training. Two levels of mental workload were set by varying the number of subtasks. EEG signals were acquired during the task. Power spectral and microstate analyses were performed on the EEG. The indices of four frequency bands (delta, theta, alpha, and beta) and four microstate classes (A–D) were calculated, changes in the frequency‐band power and microstate parameters under different mental workloads were compared, and the relationships between the two types of EEG indices were analyzed.

**Results:**

The theta‐, alpha‐, and beta‐band powers were higher under the high than under the low mental workload condition. Compared with the low mental workload condition, the high mental workload condition had a lower global explained variance and time parameters of microstate B but higher time parameters of microstate D. Less frequent transitions between microstates A and B and more frequent transitions between microstates C and D were observed during high mental workload conditions. The time parameters of microstate B were positively correlated with the delta‐, theta‐, and beta‐band powers, whereas the duration of microstate C was negatively correlated with the beta‐band power.

**Conclusion:**

EEG frequency‐band power and microstate parameters can be used to detect a high mental workload. Power spectral analyses based on frequency‐band oscillations and microstate analyses based on global brain network activation were not completely isolated during multitasking.

## Graphical abstract

This study explored the effects of simulated flight multitasking on mental workload and showed that the electroencephalography frequency‐band power and microstate parameters detected a high mental workload.

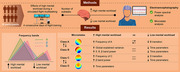

## 1 Introduction

Due to improved aircraft performance and crew resource management, air transport is considered the safest mode of transportation (Martins 2016). However, the cockpit is a multitasking work environment in which pilots must perform two or more tasks simultaneously, which can lead to a high mental workload (Martins 2016). The core requirement of multitasking management is paying timely attention to new information and integrating relevant task requirements into a task set (Hernández‐Sabaté et al. 2022). While multitasking in flight, pilots must constantly reallocate their mental resources owing to frequent attention transitions between tasks, a process of task set reconstruction (TSR) (Monsell 2003). During TSR, mental overload leads to high switching costs (Rubinstein et al. 2001). The high switch cost can manifest as an extended reaction time and increased error rate, which are considered contributing factors to many aviation accidents. Therefore, monitoring the mental workload to optimize task performance and reduce human error is significant for pilot multitasking (Kakkos et al. 2019).

By measuring dynamic physiological changes that cannot be consciously controlled, reliable data can be provided to evaluate mental workload (Charles and Nixon 2019). Research on mental workload assessment has mostly focused on various physiological signals to achieve online real‐time monitoring of pilots’ mental workload during flights (van Weelden et al. 2022). Among physiological methods, electroencephalography (EEG), considered the most effective method for detecting mental state, can directly reflect the real‐time state of the brain (Zeng et al. 2021). EEG has the characteristics of high portability, non‐invasiveness, and high temporal resolution and can provide rich information and capture subtle changes in the mental state (Bagheri and Power 2020; Chu et al. 2022). EEG characteristics have shown great potential for brain load assessment. The most researched and applied EEG index is power spectral density (PSD) (Ke et al. 2021).

EEG signals can be divided into different frequency bands based on frequency, and the most basic and common frequency bands are four bands: delta (0.5–3 Hz), theta (4–7 Hz), alpha (8–13 Hz), and beta (14–30 Hz) (Li et al. 2022b). Activity changes in different EEG frequency bands reflect the different states of the brain. Changes in theta and alpha power are sensitive indicators of mental workload; in particular, the increase in theta power in the frontal lobe and the decrease in alpha power in the parietal lobe are closely related to an increase in mental workload (Dasari et al. 2017; Mun et al. 2017; Grissmann et al. 2017).

The synchronization of theta and desynchronization of alpha reflects the activation of attentional resources. A decrease in alpha power is related to the recruitment of cognitive‐motor resources, whereas an increase in theta power is related to the mobilization of working memory and action monitoring (Gentili et al. 2018). Additionally, increased cognitive load can increase beta power (Beurskens et al. 2016). Strengthening beta activity is associated with higher order cognitive activities of behavioral control and is beneficial for improving behavioral performance (Zhao et al. 2022). The delta band seems to be an unclear indicator of the mental workload and is most common during sleep, and its power changes are mostly related to mental fatigue rather than workload (Wascher, Getzmann, and Karthaus 2016). In a simulated flight study, Yu et al. (2023) increased the mental workload by setting engine faults and found that a high mental workload led to an increase in theta power in the prefrontal lobe and a decrease in alpha power in the parietal–occipital lobe. Another simulated flight study also found an increase in theta power, in which the mental workload was differentiated on the basis of the complexity of emergency situations (Diaz‐Piedra et al. 2019). In a surveillance mission for simulated unmanned aerial vehicle control, Matthews et al. (2019) found that both negative feedback stress and high cognitive demand could lead to an increase in beta power. The above‐mentioned changes in the frequency‐band power were also found in the mental workload of multitasking. Chu et al. (2022) conducted a classical multitask attribute battery (MATB) task that can simulate multitasking of flight. An increase in mental workload increases theta and beta power but decreases alpha power. The differences in PSD are mainly concentrated in the extensive brain regions of theta, the frontal and occipital regions of alpha, and the occipital region of beta. Theta and alpha decreases were also observed in a study executing MATB in a noisy environment (Fan et al. 2022).

Although the EEG frequency‐band power is sensitive to changes in mental workload, band power changes are region‐dependent rather than global (Dasari et al. 2017). Frequency‐band power analysis independently considers the brain structure and ignores the dynamics of brain function (Spring, Tomescu, and Barral 2017). Therefore, the measurement of spontaneous EEG power is limited by two key aspects (Zanesco, Denkova, and Jha 2021): First, the measurement usually cannot distinguish between sets of brain generators that cause oscillations in the scalp electric field. Second, EEG power could not explain the large‐scale dynamics of these brain generators. However, brain function originates from a large amount of parallel processing in decentralized and distributed brain networks (Catrambone and Valenza 2023).

Cognitive processes involve the integration of various distributed brain regions responsible for different cognitive functions into large‐scale networks (Santarnecchi et al. 2017). Compared with the limited activation of brain regions that reflect basic cognitive functions, dynamic changes in large‐scale brain networks over time better represent sustained cognition (Jia et al. 2021). In addition, the interconnection between cortical regions is an influencing factor in multitasking. Therefore, neuroimaging techniques that detect spontaneous large‐scale network activities may capture the complex nature of multitasking with mental workloads.

EEG microstate analysis, one of the spatiotemporal analysis methods of multichannel EEG signals, allows for studying large‐scale distributed networks (Zappasodi et al. 2019). An EEG can be described as a series of instantaneous scalp potential topographic maps that represent the global pattern of the scalp potential that dynamically changes over time (Lehmann, Ozaki, and Pal 1987). These independent potential distribution states are defined as EEG microstates that exhibit semi‐stable and discontinuous changes, indicating that one topographic map remains stable before a rapid transition to another (Lehmann, Ozaki, and Pal 1987). Microstate analysis can be used to study the electric field in the entire brain, providing deeper insights into its temporal dynamics of the brain (Spring, Tomescu, and Barral 2017). Compared with traditional power spectral analysis, which focuses on the oscillation frequency, EEG microstate analysis retains temporal and spatial characteristics while fully utilizing multichannel information; therefore, it focuses on the spatial pattern of the voltage on the scalp over time (Cui et al. 2023). In addition, microstate analysis considers overall surface activity over a wide range of frequencies and can explore the activity of relevant cortical generators as components of neural networks (Spring, Tomescu, and Barral 2017). Therefore, time‐series‐based EEG microstates can continuously and dynamically represent the activity of different functional brain networks (Zanesco, Denkova, and Jha 2021).

EEG microstates have been proposed as building blocks that reflect information processing (Jabès et al. 2021). As markers of cognitive and psychological function, EEG microstates are regulated by task demands and sensory inputs (Seitzman et al. 2017) and can characterize perception and cognitive processing from qualitative and quantitative perspectives (Hu et al. 2023). EEG microstates can be clustered into several main components using clustering methods. The optimal number of EEG microstates depends on the specificity of the given dataset; however, four main EEG microstates are typically observed: classes A, B, C, and D (Koenig et al. 1999, 2002). The four classes of topographic maps are consistently labeled in studies of different cognitive and pathological states; therefore, they are referred to as normative or canonical microstates (Tarailis et al. 2023). Research on the function of EEG microstates has shown that the four types of microstates reflect different brain networks (Britz, Van De Ville, and Michel 2010; Custo et al. 2017). Class A was mainly related to the auditory network, whereas Class B was mainly to the visual networks. Class C is primarily related to the default mode network (DMN), although its areas partially overlap with the saliency network (Tarailis et al. 2023), whereas Class D is related to the dorsal attention network (DAN).

The relationship between microstate categories and cognitive function remains a topic of debate (Jia et al. 2021). Additionally, few studies have explored the relationship between mental workload and microstates (Chen et al. 2024; Guan et al. 2022; Jia et al. 2021; Tamano et al. 2022). Three studies used n‐back tasks as task models, but the results were inconsistent. Guan et al. (2022) and Chen et al. (2024) found that a high mental workload could decrease microstate C, whereas Tamano et al. (2022) found that the parameters of the microstate remained unchanged at different mental workload levels. In a study on creative design tasks, Jia et al. (2021) found an increase in microstates C and D during the highest cognitive load stage. Multitasking is a visual‐motor process that requires visual attention and motor execution, which differs from the processing of the n‐back and abstract psychological tasks. Among these studies, only one applied a multitasking model; however, the authors found no differences in the microstates of the two mental workloads. Chen et al. (2024) set two mental workload levels by varying the frequency of stimulus occurrence and the difficulty of joystick manipulation. The difference between the two mental workload levels may not be sufficient to induce changes in the EEG microstate because the number of concurrent tasks to be processed is the main determinant of mental workload during multitasking (Puma et al. 2018). Therefore, to explore the impact of the mental workload of multitasking on EEG microstates, this study set two levels of mental workload by changing the number of subtasks and compared the differences in EEG microstates between the two levels. In addition, EEG microstates have been extracted from broadband signals, and the connection between broadband topographic maps and brain activity in specific frequency bands remains unclear (Croce et al. 2022). Therefore, we calculated the power of specific frequency bands at two levels of mental workload to determine the relationship between microstate features and frequency‐band power.

On the basis of the above power spectral analysis of mental workload, we propose the first hypothesis that high mental workload during multitasking can lead to an increase in beta and theta power and a decrease in alpha power. Microstate B reflects the regulation of information flow intensity by the visual system (Guan et al. 2022). Microstate C is task‐negative, whereas microstate D is task‐positive (Di Muccio et al. 2023; Seitzman et al. 2017). Therefore, we propose the second hypothesis that a high mental workload can lead to an increase in microstates B and D and a decrease in microstate C. To test our hypotheses, a semi‐ecological task model (Li et al. 2022a; Li et al. 2023) was used to simulate multitasking during flights. During multitasking, four subtasks were completed simultaneously under high mental workload conditions, whereas two subtasks were completed simultaneously under low mental workload conditions. Changes in EEG frequency‐band power and microstate parameters during different mental workload conditions were compared and analyzed.

## 2 Methods

### 2.1 Participants

Thirty‐six healthy male university students (mean age, 26.86 ± 3.91) were recruited for this study. The average laterality quotient and decile of the Edinburgh Handedness Inventory (Oldfield 1971) were 83.92 ± 20.50 and 6.58 ± 3.71, respectively, which showed that the participants were all right‐handed. None of the participants had a history of neuropsychiatric disease or recent medication use.

### 2.2 Tasks

A simulated flight multitasking task (Li et al. 2023; Li et al. 2022a) was applied, which consisted of four subtasks: target tracking, meter monitoring, dot counting, and digit responses. The target‐tracking subtask required the participants to use a joystick to control a circular cursor with their right hand to track a moving flight target in the middle of the interface. The meter‐monitoring subtask required participants to monitor four meters at the bottom of the interface and press the key of the meters code once the pointer pointed to the red area. The dot‐counting subtask required participants to count a set of red dots on the left side of the interface and press the correct number key. The digit‐response subtask required participants to press the number key that matched the number displayed on the right side of the interface. The key reaction was completed by simultaneously pressing the number keys on the keyboard with the left hand and the indicator keys on the joystick with the right hand. Different subtasks corresponded to different indicator keys. Compared with the other three tasks, the digit‐response task was a secondary task with a low processing priority.

### 2.3 Protocol

**FIGURE 1:**
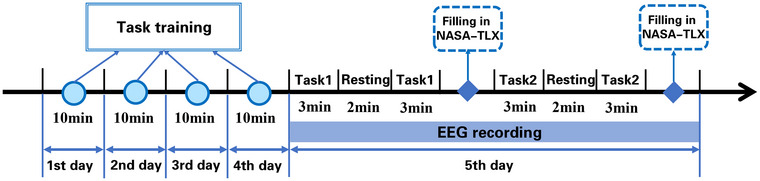
**Experiment protocol**. Tasks 1 and 2 represent two conditions of mental workload. NASA‐TLX, National Aeronautics and Space Administration‐task load index.

The experiment consisted of two stages over 5 consecutive days. The first 4 days were the training phase, and the fifth day was the testing phase. During the training phase, the participants engaged in simulated flight multitasking training for 10 min/day. The purpose of the training was to enable participants to master the task proficiently, thus minimizing the impact of learning effects on their mental workload. During the testing phase, participants completed two tasks with low and high mental workloads. In the multitasking model, two methods are used to construct the mental workload gradient (Puma et al. 2018). The first is to regulate the difficulty of subtasks. The second is to control the number of parallel subtasks. To study the influences of specific subtasks on mental workload, we established mental workload gradient by changing the number of subtasks in this study. Participants were required to complete both the target‐tracking and digit‐response subtasks simultaneously under low mental workload conditions, whereas all four subtasks were completed simultaneously under high mental workload conditions. Therefore, the difference between low and high mental workloads was the number of concurrent tasks. The order of the two conditions was counterbalanced among the participants. Each condition included two blocks, each lasting 3 min. To reduce the impact of fatigue, there was a 2‐min resting period between the two blocks in each condition. The National Aeronautics and Space Administration‐task load index (NASA‐TLX) scale was completed after each task. The NASA‐TLX is a subjective mental workload assessment scale that includes six dimensions: mental demand, physical demand, temporal demand, performance level, effort level, and frustration level (Hart and Staveland 1988). In addition to using scales to record subjective feelings, the EEG of the participants was collected throughout the entire process of the task. The experimental protocol is illustrated in Figure 1.

### 2.4 EEG Acquisition and Analysis

#### 2.4.1 EEG Data Acquisition

Thirty‐one electrodes (Fp1/2, Fpz, F7/8, F3/4, Fz, FC5/6, FC1/2, T7/8, C3/4, Cz, CP5/6, CP1/2, CPz, P7/8, P3/4, Pz, POz, O1/2, Oz) EEG signals were recorded using ANT eego mylab device (ANT Neuro, Germany). Silver/silver chloride (Ag/AgCl) electrodes were placed according to the international standard 10–20 system.

The impedances of all electrodes were maintained below 5 kΩ. All the signals were referenced online to the CPZ electrode and recorded at a sampling frequency of 1000 Hz. A bandpass filter 0.3–50 Hz was applied during signal acquisition.

#### 2.4.2 EEG Data Preprocessing

MATLAB (Version 2015b, MathWorks) and the EEGLab toolbox (Version 14, http://sccn.ucsd.edu/eeglab/) (Delorme and Makeig 2004) were used for EEG preprocessing. The EEG data were down‐sampled offline to 250 Hz. Signals were filtered with a 50 Hz notch filter and a 1–40 Hz bandpass filter. Data with amplitudes >100 µV were labeled as artifacts and excluded, and further visual inspection was conducted to remove residual artifacts (Kim et al. 2021). Independent component analysis (ICA) was performed to detect and remove components related to other artificial activities, such as eye movement, muscle activities, and heartbeat (James and Hesse 2005). In addition, the data were referenced to a common average. Finally, the preprocessed data were segmented into 2 s epochs for further analyses.

#### 2.4.3 EEG Power Spectral Analysis

Fourier transform was performed on the processed data to transfer the EEG from the time domain to the frequency domain. According to the frequency range, EEG was divided into four bands: delta (0.5–3 Hz), theta (4–7 Hz), alpha (8–13 Hz), and beta (14–30 Hz) (Li et al. 2022b). The periodogram method was used for power spectrum calculation. For a sequence *x* (*n*) of length *N*, whose Fourier transform is *x* (*k*), the power at frequency *k* was expressed as follows:

(1) $$P\left(k\right)=\frac{1}{N}{\left|X\left(k\right)\right|}^{2}$$

Then the average was taken:

(2) $$PBi=\frac{1}{\left|Bi\right|}\times \sum_{k\in Bi}P\left(k\right)$$

where Bi is the set of frequency points included in the ith frequency band, P(k) the power of frequency k, and PBi the average power of frequency‐band Bi.

The average power at all frequency points within the four different frequency bands was extracted.

#### 2.4.4 EEG Microstate Analysis

For EEG microstate analysis, the EEGlab plug‐in Microstate Analysis toolbox (Version 0.3, Thomas König) was used to preprocess the data (Poulsen et al. 2018). First, the global field power (GFP), reflecting the standard deviation of the voltage across all electrode channels on the scalp at a certain moment, was calculated. Owing to the high stability and signal‐to‐noise ratio of topographies with higher GFP, only topographies located at the peak of GFP were extracted and subjected to *k*‐means clustering analysis (Koenig et al. 2002). The polarity of the topography was ignored. Canonical microstates are topographical representations based on a large amount of prior knowledge, which are beneficial for comparing microstate differences across groups and cognitive states (Kim et al. 2021). Therefore, referring to relevant research (Chen et al. 2024), we set *k* = 4 to facilitate comparison with similar studies. Four types of topographic maps were extracted for each participant to explain the maximum variance in all topographic maps. The four topographic maps of each subject were clustered into the grand mean template and labeled as microstates A, B, C, and D. Then, a back‐fitting step was implemented to calculate the spatial correlation coefficient between the original topographic maps of each subject at the GFP peaks and the grand mean template. Each original topographic map was labeled with the class of EEG microstates with the highest Pearson correlation coefficient. Subsequently, the EEG signals were converted into a time series of four classes of EEG microstates.

The total global explained variance (GEV) of the four microstates and the three dynamic parameters (duration, occurrence, and coverage) of each microstate were calculated under two mental workload conditions (high and low). The duration is the mean time for each microstate to remain stable. Occurrence was defined as the average number of occurrences per second for each microstate. The coverage is the percentage of each microstate‐time relative to the total time. The transition probability, the percentage of transitions from one microstate to another, was also calculated to assess the microstate syntax of the microstate sequence.

### 2.5 Statistical Analysis

Paired‐sample *t*‐tests with a within‐subject design were used to test whether the effects of mental workload (low and high) on NASA‐TLX scores, task performance, average power of EEG frequency bands, and EEG microstate parameters were statistically significant. One‐way repeated measures analysis of variance (ANOVA) with the factors “training times” tested whether the effects of training on task performance were statistically significant. Fisher protected least significant difference (Fisher PLSD) was applied to compare task performance on 2 consecutive days during the training period. The link between EEG frequency‐band characteristics and EEG microstate parameters was tested using Pearson's correlation. The degree of correlation was defined on the basis of the correlation coefficient: *R* > 0.70 is strong, 0.50< *R* < 0.70 is moderate, and 0.30 < *R* < 0.50 is weak (Kahya et al. 2022). Multiple comparisons of EEG frequency‐band power for each electrode and task performance for different training times were corrected using the false discovery rate (FDR) method. SPSS software (version 26.0) was used for statistical analysis, and significance was set at *p* < 0.05.

## 3 Results

### 3.1 Task Performance

#### 3.1.1 Training Effect on Task Performance

**FIGURE 2:**
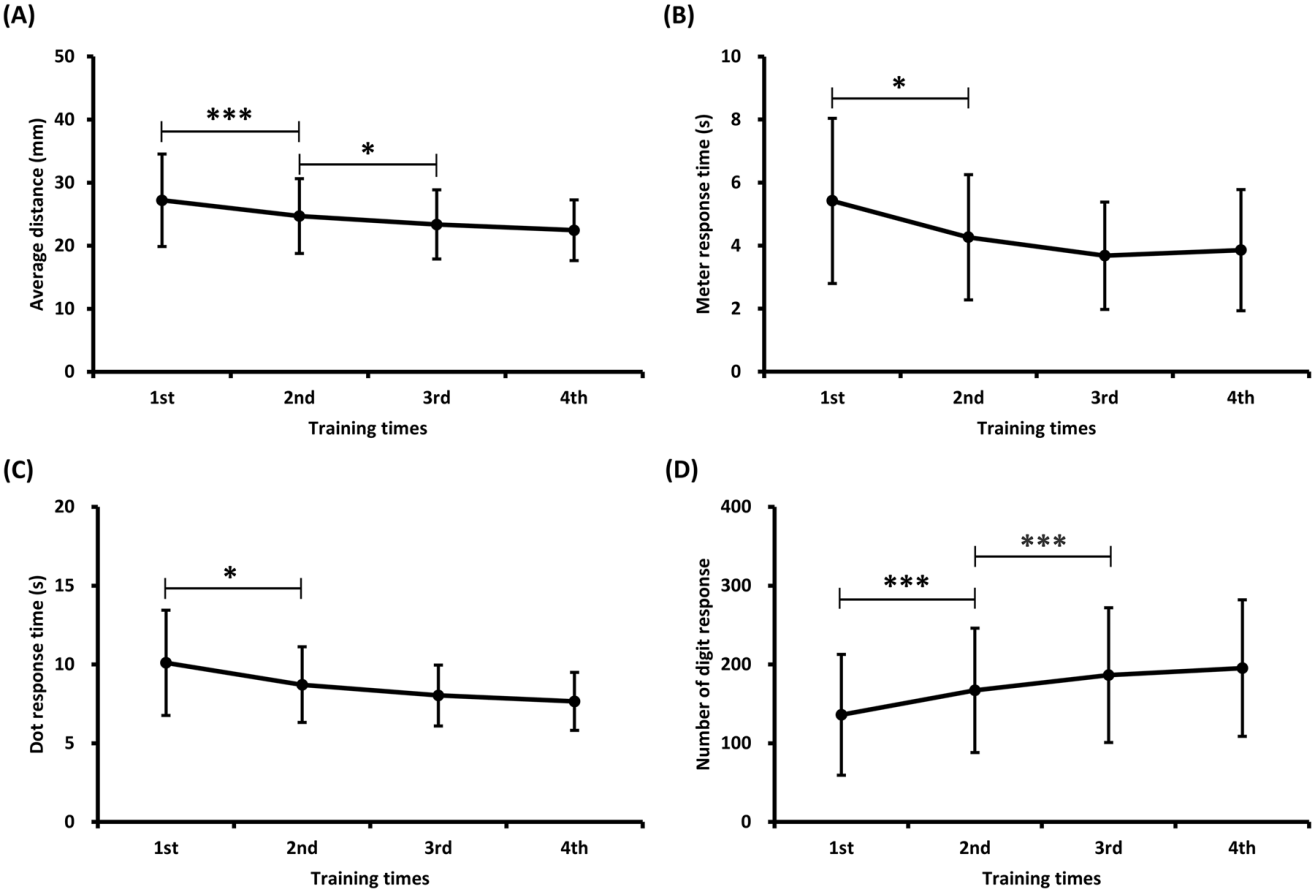
**Task performance at different training times**. (A) Average distance, (B) meter response time, (C) dot response time, and (D) number of numeral responses. The error bars represent the standard deviation. *p < 0.05, ***p < 0.001.

Figure 2 illustrates task performance during the training phase. The average distance between the circular cursor and the target was the performance of the target‐tracking task. A shorter distance indicates better performance. The performance of the meter monitoring and dot‐counting tasks was the response time to the stimuli. Digit‐response task performance was assessed by the number of responses. One‐way repeated measures ANOVA showed significant differences in training effects on tracking distance (*F* = 23.98, *p* < 0.001, *η* ^2^_p_ = 0.41), meter response time (*F* = 9.18, *p* < 0.001, *η* ^2^_p_ = 0.21), dot response time (*F* = 12.80, *p* < 0.001, *η* ^2^_p_ = 0.27), and digit‐response number (*F* = 35.28 *p* < 0.001, *η* ^2^_p_ = 0.50). As shown in Figure 2A, when comparing two adjacent training times, the tracking distance for the second time was smaller than that for the first time (*t* = 4.29, *p*‐corrected < 0.001, Cohen's *d* = 0.38), and the tracking distance for the third time was smaller than that for the second time (*t* = 2.56, *p*‐corrected = 0.022, Cohen's *d* = 0.23). As shown in Figure 2B, the second meter response time was shorter than the first (*t* = 2.90, *p*‐corrected = 0.019, Cohen's *d* = 0.50). As shown in Figure 2C, the dot response time in the second session was shorter than that in the first session (*t* = 2.74, *p*‐corrected = 0.030, Cohen's *d* = 0.48). As shown in Figure 2D, the digit‐response number of the second time was higher than that of the first time (*t* = −5.13, *p*‐corrected < 0.001, Cohen's *d* = 0.40), and the digit‐response number of the third time was higher than that of the second time (*t* = −4.57, *p*‐corrected < 0.001, Cohen's *d* = 0.24).

#### 3.1.2 Mental Workload Effect on Task Performance

Table 1 lists the task performances during the testing phase. Compared with the low mental workload, in the high mental workload condition, the average tracking distance increased (*t* = −7.79, *p* < 0.001, Cohen's *d* = 0.71), whereas the number of digit responses decreased (*t* = 16.29, *p* < 0.001, Cohen's *d* = 1.10). The meter and dot response times in high mental workload were 3.82 ± 1.89 and 7.91 ± 1.94 s, respectively.

**TABLE 1:** Task performance in different mental workload conditions.

Feature	Low mental workload	High mental workload
Average distance (mm)	19.91 ± 3.49	22.81 ± 4.65***
Number of digit response	295.92 ± 87.24	199.81 ± 88.03***
Meter response time (s)	None	3.82 ± 1.89
Dot response time (s)	None	7.91 ± 1.94

***p < 0.001, as compared with low mental workload.

### 3.2 Subjective Scores

**FIGURE 3:**
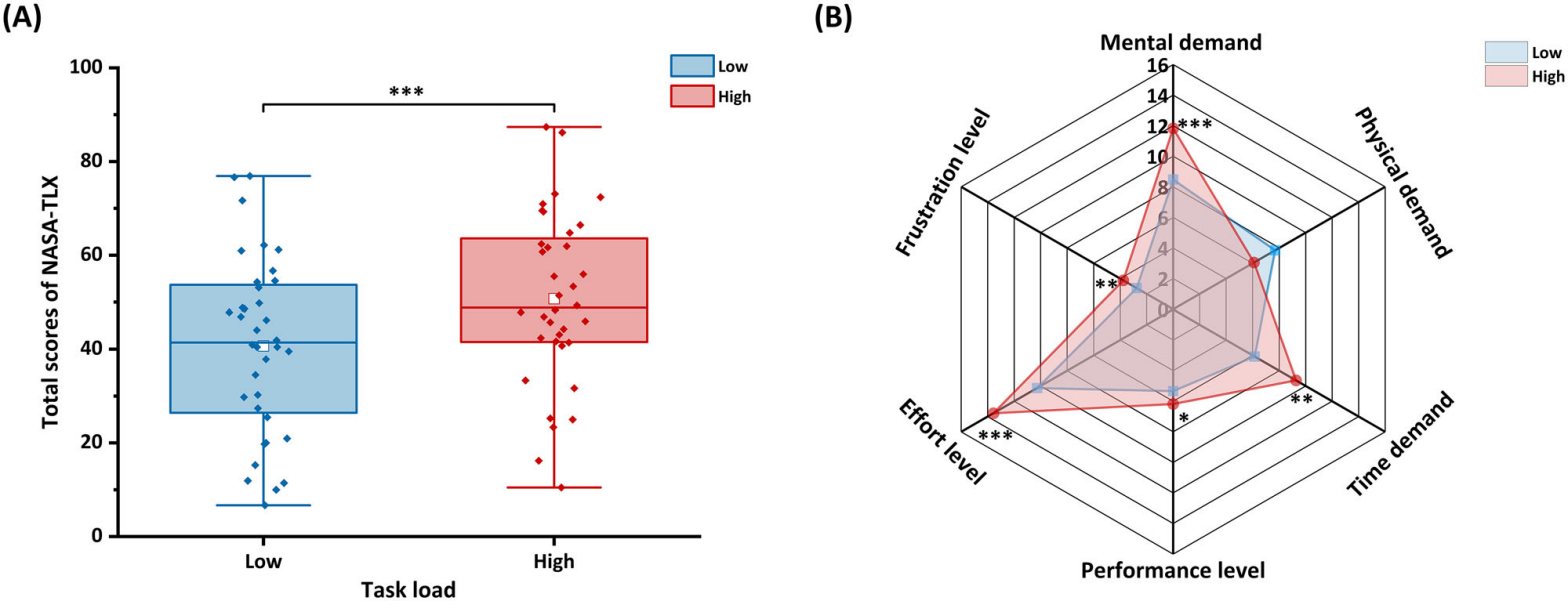
**NASA‐TLX scores in different mental workloads**. (A) Total scores and (B) scores of six dimensions. The lower and upper ends of the box represent the first quartile (Q1) and third quartile (Q3), respectively, and the difference between Q3 and Q1 is the interquartile range (IQR). The horizontal lines and white squares inside the box represent the median and mean values. The lower and upper ends of the error bars are Q1 − 1.5IQR and Q3 + 1.5IQR. *p < 0.05, **p < 0.01, ***p < 0.001. NASA‐TLX, National Aeronautics and Space Administration‐task load index.

As shown in Figure 3A, the total NASA‐TLX score in high mental workload condition was higher than that in low mental workload condition (*t* = −8.26, *p* < 0.001, Cohen's *d* = 0.54). Figure 3B shows the scores for the six dimensions. Compared to the low mental workload, in the high mental workload condition, the scores of mental demand (*t* = −4.06, *p* < 0.001, Cohen's *d* = 0.43), time demand (*t* = −3.82, *p* = 0.001, Cohen's *d* = 0.63), performance level (*t* = −2.27, *p* = 0.030, Cohen's *d* = 0.23), effort level (*t* = −4.59, *p* < 0.001, Cohen's *d* = 0.54), and frustration level (*t* = −2.84, *p* = 0.007, Cohen's *d* = 0.36) were increased.

### 3.3 EEG Frequency Bands

**FIGURE 4:**
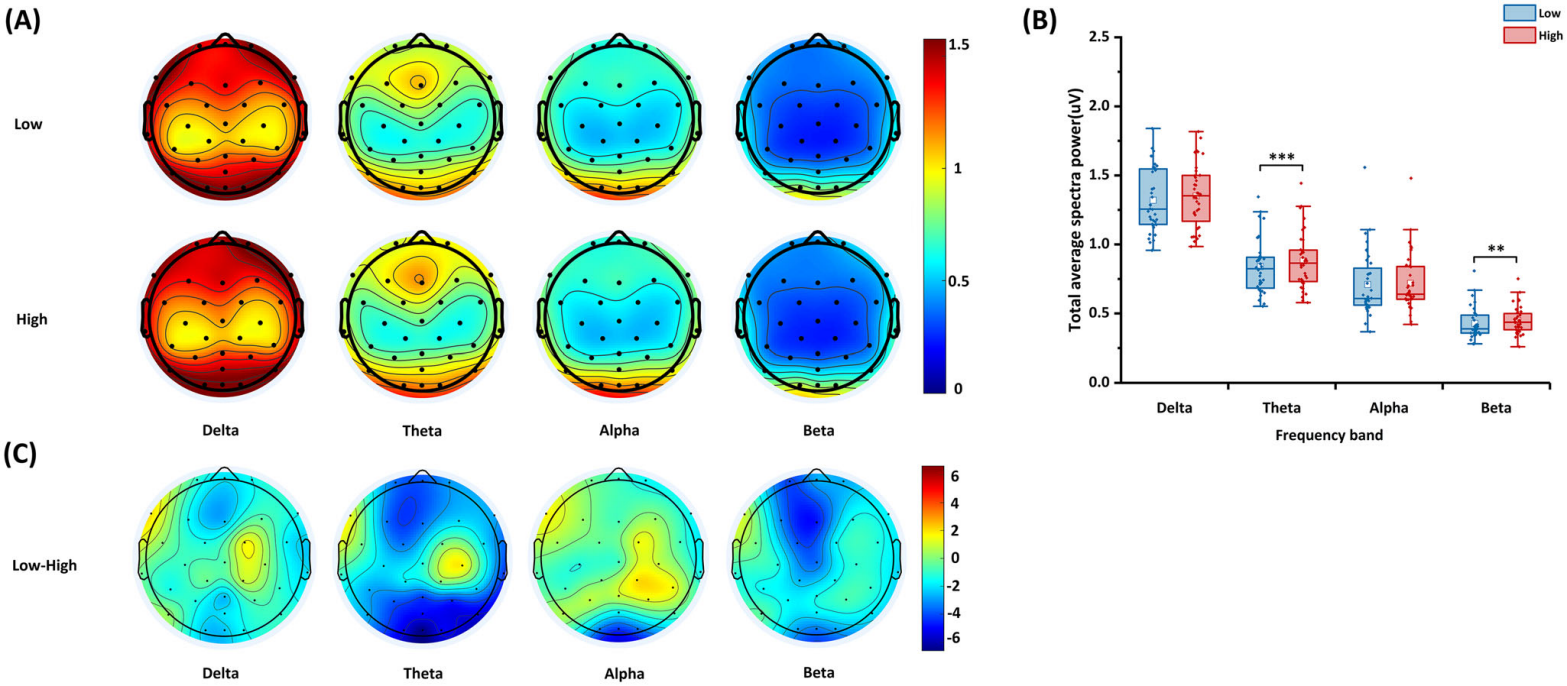
**Frequency‐band power in different mental workloads**. (A) Topographies of frequency‐band power, (B) statistical graph of total average spectra power, and (C) distribution graph of statistical values. The lower and upper ends of the box represent the first quartile (Q1) and third quartile (Q3), respectively, and the difference between Q3 and Q1 is the interquartile range (IQR). The horizontal lines and white squares inside the box represent the median and mean values. The lower and upper ends of the error bars are Q1 − 1.5IQR and Q3 + 1.5IQR. **p < 0.01, ***p < 0.001.

Figure 4A,B shows the average power of the EEG frequency bands under different mental workload conditions. Compared with the low mental workload condition, in the high mental workload condition, the total average power of the theta band (*t* = −4.49, *p* < 0.001, Cohen's *d* = 0.20) and beta band (*t* = −3.13, *p* = 0.003, Cohen's *d* = 0.19) increased. Figure 4C shows the *t* values for comparing each electrode under the two conditions. The theta‐band power of the high mental workload was higher than that of the low mental workload for 18 electrodes. The alpha‐band power of the high mental workload was higher than that of the low mental workload for the three electrodes. The beta‐band power of the high mental workload was higher than that of the low mental workload for 10 electrodes. For details of the results, see Table S1.

### 3.4 EEG Microstates

#### 3.4.1 EEG Microstate Temporal Parameters

**FIGURE 5:**
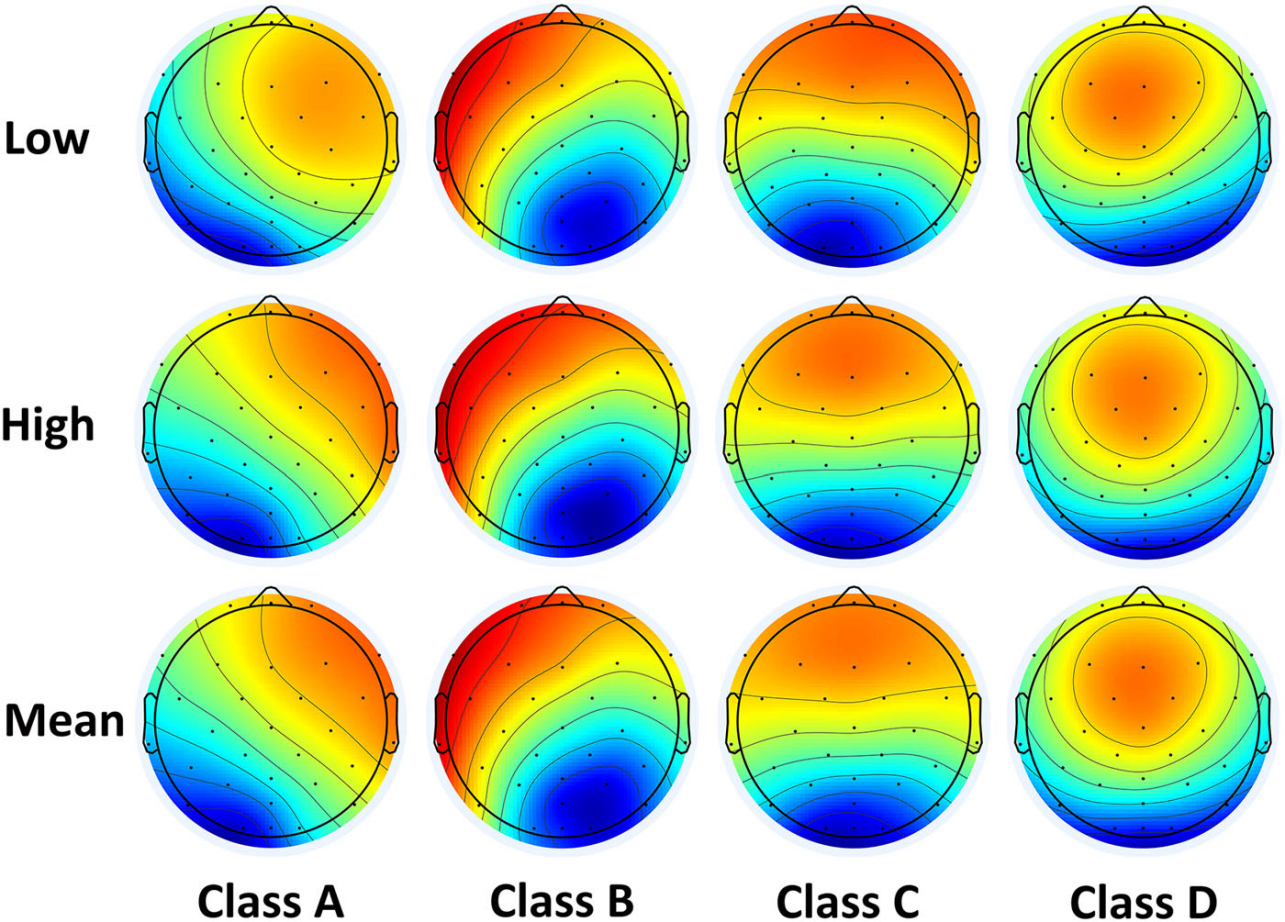
**Topographies of the four classes of microstates in different conditions**.

**FIGURE 6:**
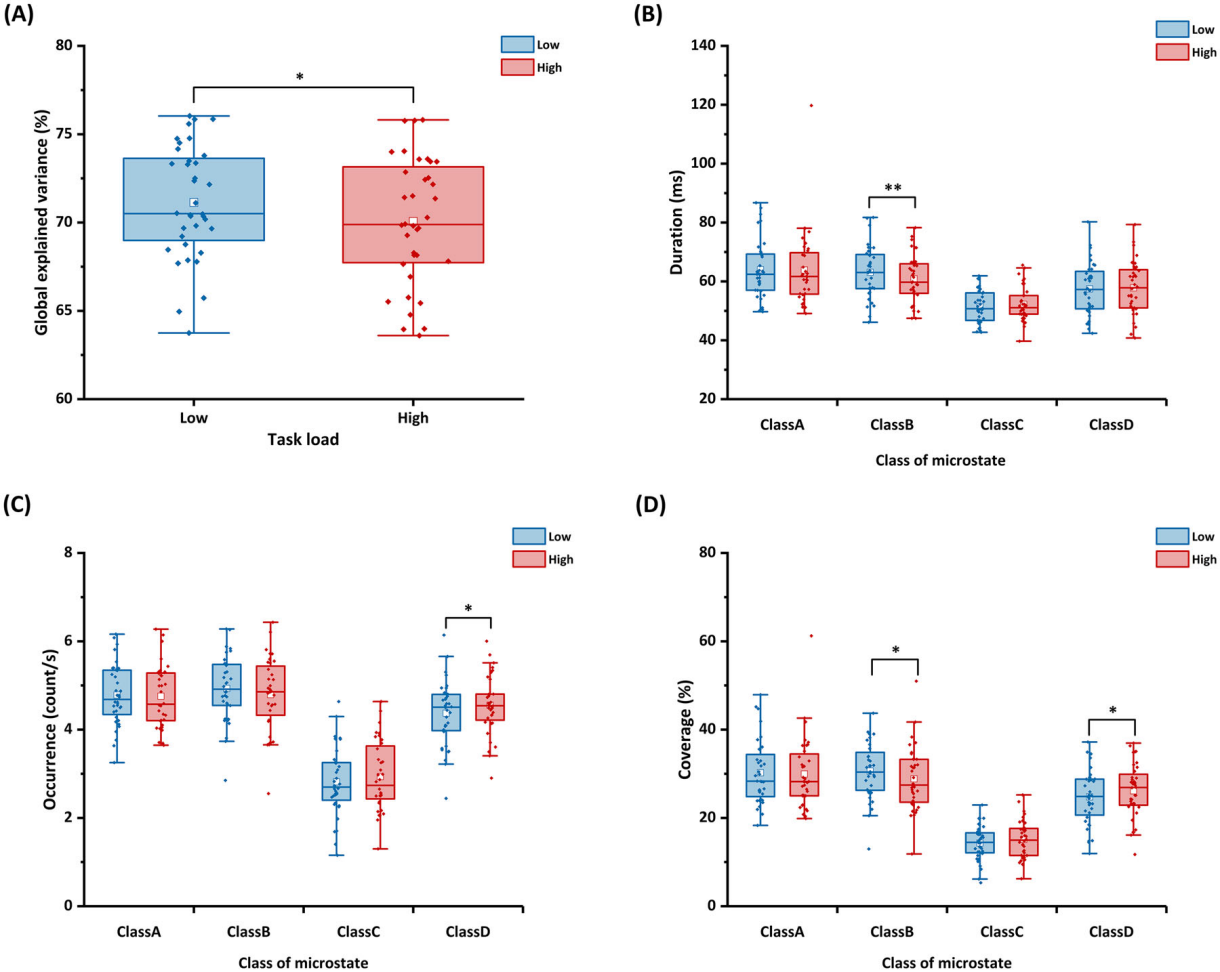
**Microstate temporal parameters results**. (A) The global explained variance, (B) the mean duration, (C) the occurrence, and (D) the coverage. The lower and upper ends of the box represent the first quartile (Q1) and third quartile (Q3), respectively, and the difference between Q3 and Q1 is the interquartile range (IQR). The horizontal lines and white squares inside the box represent the median and mean values. The lower and upper ends of the error bars are Q1 − 1.5IQR and Q3 + 1.5IQR. *p < 0.05, **p < 0.01.

Figure 5 shows the topographies of the four EEG microstate classes. Class A is displayed as a right frontal‐to‐left posterior configuration, Class B as a left frontal‐to‐right posterior configuration, Class C as a symmetric anterior‐to‐posterior configuration, and Class D as a frontocentral configuration. Figure 6 shows the temporal parameters of the four microstates under two mental workload conditions. As shown in Figure 6A, the GEV was significantly lower in the high mental workload condition than in the low mental workload condition (*t* = 2.47, *p* = 0.018, Cohen's *d* = 0.32). As shown in Figure 6B, the mean duration for Class B in the high mental workload condition was lower than that in the low mental workload condition (*t* = 2.87, *p* = 0.007, Cohen's *d* = 0.26). As shown in Figure 6C, the occurrence of class D in the high mental workload condition was higher compared with the low condition (*t* = −2.35, *p* = 0.024, Cohen's *d* = 0.26). As shown in Figure 6D, compared with the low mental workload condition, in the high condition, the coverage of class B (*t* = 2.28, *p* = 0.029, Cohen's *d* = 0.26) decreased, whereas that of class D (*t* = −2.72, *p* = 0.010, Cohen's *d* = 0.23) increased.

#### 3.4.2 EEG Microstate Syntax

**FIGURE 7:**
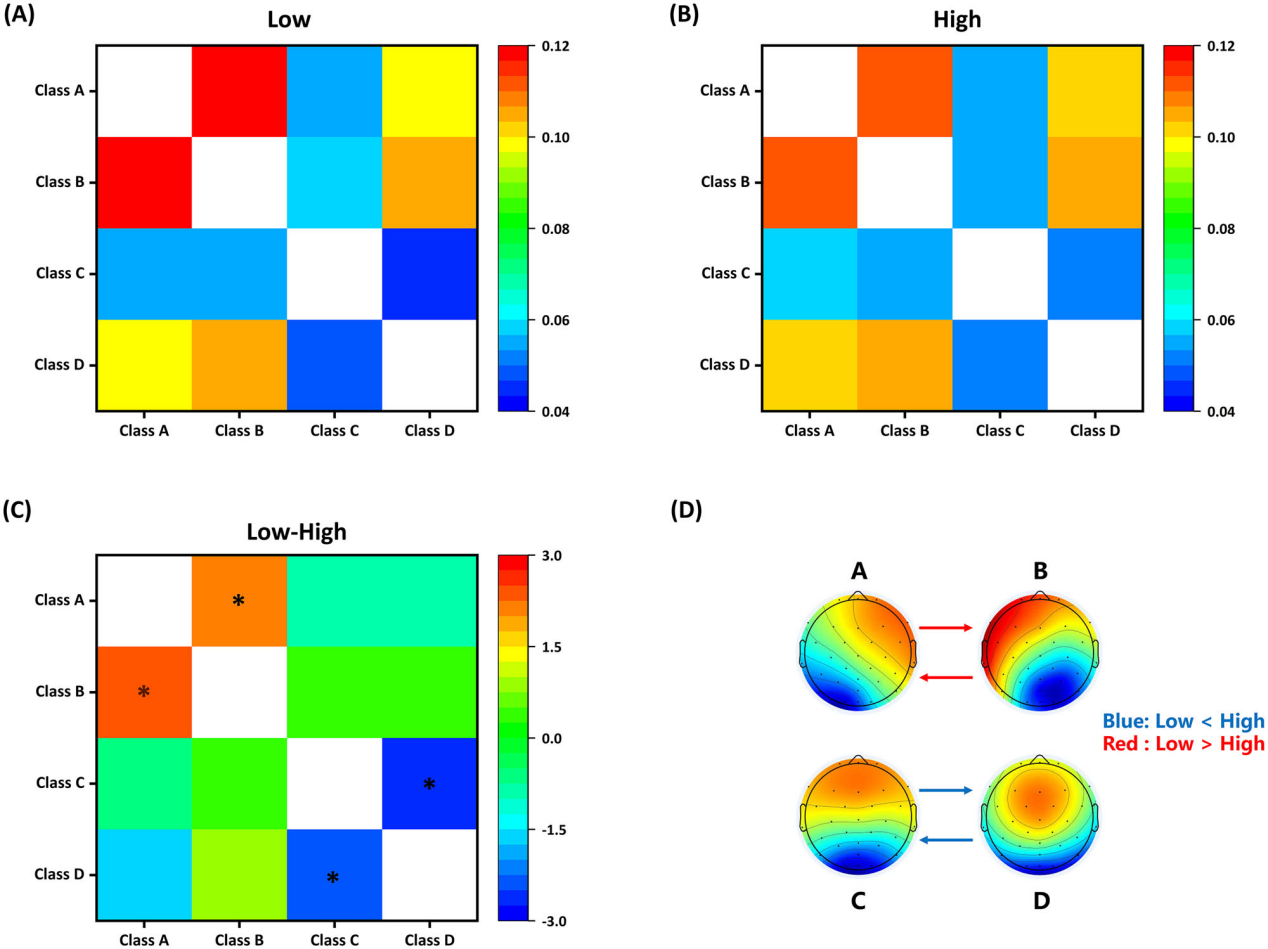
**Microstate syntax results**. (A) Transition probabilities in the low mental workload condition, (B) transition probabilities in the high mental workload condition, (C) distribution graph of statistical values, and (D) the significant transition probabilities between the two conditions. *p < 0.05.

Figure 7 shows the transition probabilities between the microstates. The transition probabilities for the two mental workload conditions are shown in Figure 7A,B. Figure 7C,D shows *t* values in the comparison of transition probabilities and the significant differences between the two conditions. Compared with the low mental workload condition, in the high condition, the transition probability from A → B (*t* = 2.42, *p* = 0.021, Cohen's *d* = 0.32) and B → A (*t* = 2.23, *p* = 0.032, Cohen's *d* = 0.29) decreased, whereas C → D (*t* = −2.38, *p* = 0.023, Cohen's *d* = 0.32) and D → C (*t* = −2.60, *p* = 0.013, Cohen's *d* = 0.34) increased.

### 3.5 Correlation Analysis

**FIGURE 8:**
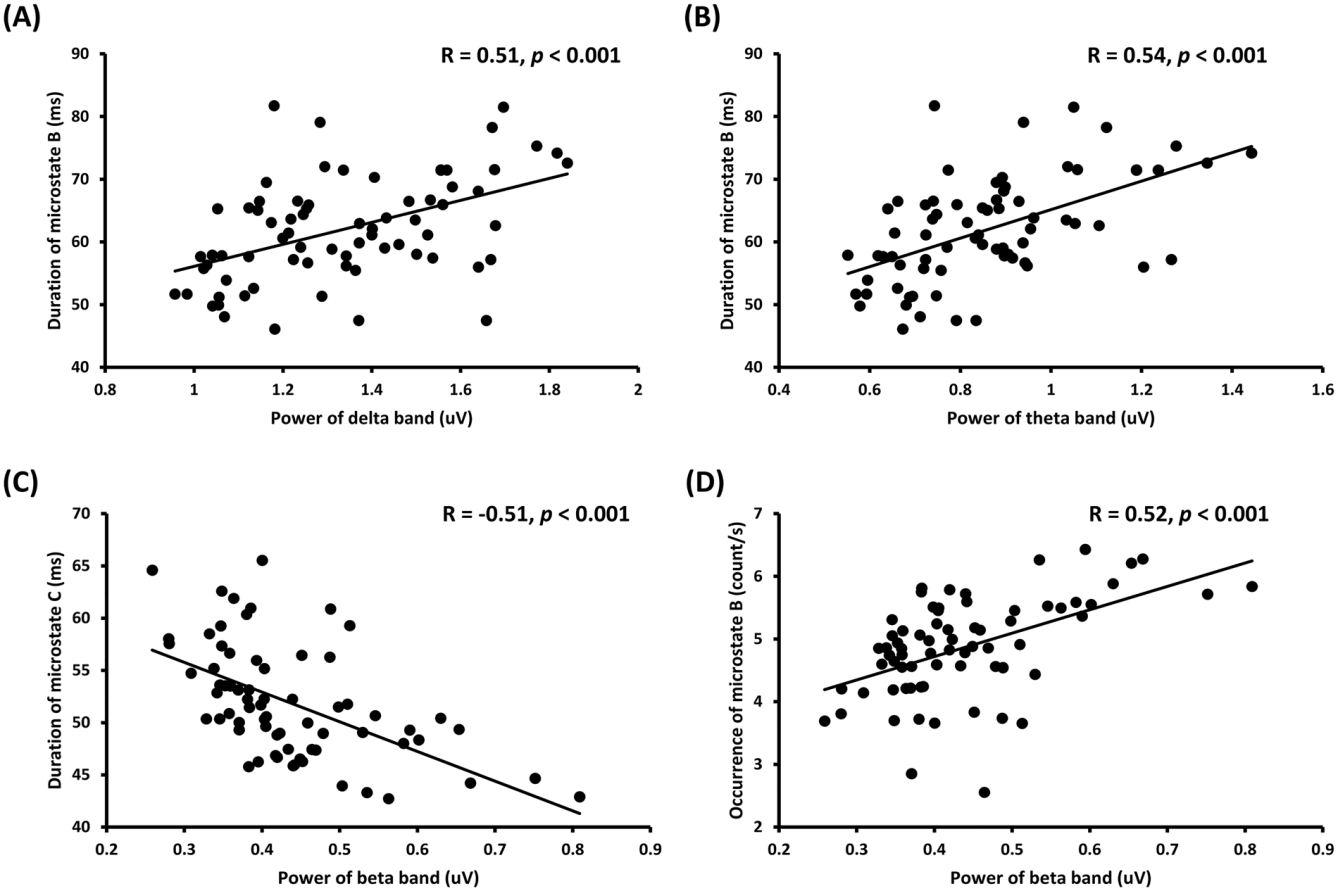
**Scatterplot with regression lines**. Regression plots of the correlation between the (A) duration of microstate B and delta‐band power, (B) duration of microstate B and theta‐band power, (C) duration of microstate C and beta‐band power, and (D) occurrence of microstate B and beta‐band power.

The analysis showed significant moderate correlations between the frequency‐band power and microstate parameters in the four tests. Figure 8A,B shows the positive correlations between the duration of microstate B and delta‐band power (*R* = 0.51, *p* < 0.001) and theta‐band power (*R* = 0.54, *p* < 0.001). Figure 8C,D shows a negative correlation between the duration of microstate C and beta‐band power (*R* = −0.51, *p* < 0.001) and a positive correlation between the occurrence of microstate B and beta‐band power (*R* = 0.52, *p* < 0.001). The other correlations were weak (*R* < 0.40) or not significantly different (*p* > 0.05). Detailed results are provided in Table S2.

## 4 Discussion

This study explored the sensitivity of EEG microstate detection to mental workload leveled by the number of subtasks and the correlation between EEG microstate and frequency‐band power in multitasking. To eliminate the impact of learning effects, a 4‐day continuous training was conducted before the mental workload testing phase. There were no significant differences in the performance of the four subtasks between the last two training sessions, indicating that the proficiency of the subjects in the task reached a plateau. This ensured that the changes in various indices during the testing phase were mostly caused by the mental workload.

During the testing phase, a high mental workload led to poorer task performance, which manifested as an increase in the tracking distance and a decrease in the number of digit responses. This decrease in performance can be explained using the theory of multiple‐resource model (Wickens et al. 2016). This model contains four dimensions: processing stages (perception and responding cells), processing codes (spatial and verbal), perceptual modalities (visual and auditory), and visual channels (focal and ambient). Any task occupies one or more units (a category in one dimension) of multiple resources, and multiple tasks can interfere with each other due to resource competition because they occupy the same units. In this study, resource competition under high mental workloads decreased the resources used for each subtask, leading to decreased performance. In addition, the decline in task performance may be due to switching costs caused by frequent task switching (Monsell 2003), and the more complex the task, the greater the switching costs (Rubinstein et al. 2001). The decline in performance was also reflected in an increased score in the performance and frustration levels of the NASA‐TLX, which indicated that the subjects were dissatisfied with their performance during multitasking.

In the EEG power spectral analysis, the mental workload impacted theta, alpha, and beta powers. The theta power increased in most brain regions, consistent with previous studies (Chu et al. 2022; Fan et al. 2022; Ke et al. 2021). An increase in theta power is an indicator of increased working memory requirements. There are generators of theta activity in the medial prefrontal cortex (PFC), and working memory is regulated through top‐down signals from the PFC to posterior brain regions (Dimitriadis et al. 2016). Sauseng et al. (2010) proposed theta activity as an integrated brain mechanism that coordinates several brain regions involved in working memory. In addition to working memory, theta activity is related to executive control and focused attention, and several cortical regions are involved in the processing of complex tasks (Diaz‐Piedra, Sebastián, and Di Stasi 2020). In addition, increased theta power reflects an overload of attention control and information encoding in brain networks (Mun et al. 2017). Therefore, the present study observed an increase in the theta power under high mental workload conditions in the frontal, parietal, temporal, and occipital lobes. As an indicator of increased alertness (Li et al. 2022b), beta activity was enhanced in this study, consistent with the previous research (Fabre et al. 2023), in which the beta power in multitasking alarm response and mental arithmetic tasks was higher than that in single‐tasking alarm response tasks. Beta‐phase synchronization was found in frontoparietal coupling during attentional tasks, indicating that beta activity is the mechanism of spreading attention arousal (Kamiński et al. 2012). In addition, beta activity can be a carrier of attention activation in the visual system; therefore, during high‐attention‐demanding tasks, an increase in beta power is beneficial for promoting sensitivity to visual input (Gola et al. 2013). These mechanisms explain the increase in the beta power in the frontal and occipital lobes observed in the present study. Unlike the changes in theta and beta power, the increase in alpha power was contrary to this hypothesis.

The stimulation of attentional resources is related to the desynchronization of the alpha band, and several studies on multitasking mental workload have found that the power of the alpha band decreases with an increase in mental workload (Chu et al. 2022; Fan et al. 2022; Ke et al. 2021; Shaw et al. 2018), inconsistent with our results. However, Puma et al. (2018) proposed that alpha synchronization and desynchronization may be responsible for two mechanisms of working memory maintenance. Alpha desynchronization is related to processing task‐related information, whereas alpha synchronization is related to suppressing interference information. The authors found that as the number of concurrent subtasks increased, the alpha power in the occipital lobe increased and considered that this result reflected the inhibition of irrelevant processing. In a dual‐task study that induced mental workload by synchronously performing spelling and dichotic listening tasks, an increase in alpha power in the occipital lobe during high mental workloads was also found (Käthner et al. 2014). To suppress the extraction of task‐irrelevant information, frequent task switching can lead to alpha synchronization (Puma et al. 2018). A study of an actual flight evaluated mental workload during the takeoff, airwork, and landing stage (Di Stasi et al. 2015). The alpha power increases during the takeoff and landing stages, where switches between tasks often occur. In addition, external visual information is stored in the posterior brain area through alpha rhythms (Dimitriadis et al. 2016), which explains why changes in alpha power were observed mainly in the occipital lobe in the above research and the present study. Therefore, an increase in the mental workload during multitasking can lead to different changes in alpha power. A high mental workload induced by increasing the frequency of task‐related information led to a decrease in alpha power. In contrast, a high mental workload induced by increasing the number of subtasks led to an increase in alpha power.

The power at a single electrode confuses the oscillation contributions of different brain generators observed on the scalp, whereas EEG microstates can characterize the temporal dynamics of different functional brain networks, which better explains the neural basis of sustained attention (Zanesco, Denkova, and Jha 2021). In the EEG microstate analysis, the mental workload had an impact on the total GEV, parameters of microstate B, and parameters of microstate D. Compared with low mental workload conditions, the total GEV decreased under high mental workload conditions. A probable explanation for this is that multiple visual stimuli must be tracked under high mental workload conditions, and visual stimuli increase the number of distributed processes in the brain, decreasing the GEV of the four canonical microstates (Seitzman et al. 2017). An alternative explanation is that error‐related execution can lead to a decrease in GEV (Walia et al. 2022), consistent with the observed decrease in performance under high mental workload conditions. Among the microstate‐time parameters, only the change in microstate D was consistent with our hypotheses. A high mental workload increased the time parameters of microstate D, consistent with studies showing that increased attention leads to an increase in microstate D (D'Croz‐Baron et al. 2020; Deolindo et al. 2021; Jia et al. 2021; Seitzman et al. 2017). Microstate D is mainly related to the DAN of the frontal and parietal lobes (Tarailis et al. 2023). The dorsal attention system is a functional system activated by attentional tasks (Seitzman et al. 2017) and is responsible for top‐down goal‐oriented attentional control (Bagdasarov et al. 2024). Therefore, microstate D is mainly related to execution processes, such as working memory, cognitive control, and redirected attention (Tarailis et al. 2023). Microstate D is also related to the activation of the inferior parietal lobe, which is beneficial for integrating relevant stimuli and sensory movements during task execution, thereby achieving robust motor execution (Penalver‐Andres et al. 2022). In addition, negative emotions can increase microstate D (Hu et al. 2023), and microstate D plays a role in error handling (Bagdasarov et al. 2024). In this study, multitasking under high mental workload conditions required subjects to track multiple information stimuli simultaneously, switch attention promptly, make corresponding responses, and execute actions under time pressure. During this process, excessive task demands increase the error rates of tasks and the frustration of the subject. Therefore, attention, error handling, and emotions collectively explain the increase in microstate D during a high mental workload.

The potential neural sources of microstate B are the activation of the occipital cortex and visual network (Tarailis et al. 2023). According to this hypothesis, an increase in visual stimuli in high mental workload conditions should increase microstate B. However, the observed decrease in microstate B was contrary to this hypothesis. The visual channel dimension of the multiple‐resource model (Wickens et al. 2016) may explain this contradiction. An increase in the number of subtasks during high mental workload conditions increased visual stimulation. However, when the subjects performed a specific subtask, only the visual stimulus of that subtask was focal vision; the other visual stimuli were ambient vision. For example, when completing the dot‐counting subtask, subjects focused their attention on the visual stimuli of the dot while ignoring other visual stimuli. As shown in the results, the average time for this process was approximately 8 s, during which only dot visual stimuli were processed. Therefore, the visual stimuli that were effectively processed during high mental workload conditions were reduced, leading to decreased visual perception processing. Jia et al. (2021) compared the EEG microstates of different task stages in creative design tasks and found that microstate B decreased during high cognitive load stages, which the authors explained as reflecting attention concentration. In addition, Antonova et al. (2022) found that less effort was associated with a longer duration of microstate B during the visualization process, consistent with the trend of microstate B observed in this study.

Unlike microstates B and D, microstate C exhibited no significant changes. Microstate C is believed to have generators in the precuneus and posterior cingulate cortex and is related to the DMN (Custo et al. 2017), which is more active during the resting state (Michel and Koenig 2018). Microstate C is also considered to be related to the saliency network (Britz, Van De Ville, and Michel 2010), but the related results may be caused by the inappropriate selection of the number of clusters to be extracted (Tarailis et al. 2023). Therefore, the cognitive function associated with microstate C remains unclear. Nonetheless, many studies (Bréchet et al. 2019; Chen et al. 2024; Kim et al. 2021; Seitzman et al. 2017; Zappasodi et al. 2019; Zhang et al. 2024) have reported that microstate C is task‐negative and that cognitive tasks decrease microstate C. Therefore, we assumed that a high mental workload would decrease microstate C. However, the results for microstate C were not as expected, possibly because of the offsetting effect. According to the selective attention and cognitive control theory proposed by Lavie et al. (2004), a high mental workload can consume information processing resources, decreasing the ability to actively suppress interfering information. A reduced ability to filter irrelevant information is associated with greater activity in the posterior cingulate cortex of the DMN (Milton et al. 2007), and more activation of the DMN can lead to an increase in microstate C and a decrease in task performance (Kim et al. 2021). Therefore, we infer that the direct effect of a high mental workload leads to a decrease in microstate C; however, a decrease in the ability to filter irrelevant information caused by a high mental workload increases microstate C. These two effects canceled each other and resulted in no significant change in microstate C. Further research is needed to validate this explanation.

In addition to the time parameters of the microstate, this study investigated microstate syntax under different mental workload conditions. Less frequent transitions between microstates A and B and more frequent transitions between microstates C and D were observed under high mental workloads. Microstates A and B are related to the brain network involved in sensory processing, whereas C and D are related to the brain network that supports higher order cognitive function (Jabès et al. 2021). The lower transitions between A and B reflect insufficient switching between the auditory and visual networks, whereas the higher transitions between C and D reflect more sufficient switching between the DMN and DAN (Cui et al. 2021). This change may be related to the effects of distracting stimuli. Multitasking can lead to greater distraction from irrelevant information (Zhang et al. 2024). In this study, when the tracking distance of the target‐tracking subtask exceeded 20 mm, an alarm sound was emitted, which was an auditory distraction stimulus for the other subtasks being executed. In a study exploring the impact of auditory distractions on visual tasks, Korn et al. (2021) found that auditory distraction suppressed the transition between auditory and visual networks, leading to a decrease in the probability of transition between A and B. In contrast, an increased transition probability between C and D was found, indicating that auditory distraction leads to alternating activation of the DMN and DAN (Korn et al. 2021). In addition, static and dynamic functional connections between the DMN and DAN are stronger during multitasking than single‐task processing (Lam, Vartanian, and Hollands 2022). This indicates that higher order cognitive tasks require flexible coupling activities between the DMN and the DAN (Kim et al. 2021). Therefore, sufficient transition and balance between higher order networks are crucial for performing working memory tasks (Tamano et al. 2022) and are beneficial for regulating performance (Bagdasarov et al. 2024).

Furthermore, we explored the relationship between the microstate features and frequency‐band power during multitasking. Spring, Tomescu, and Barral (2017) found no significant relationship between EEG microstates and frequency‐band power in an endurance exercise task. This lack of correlation indicates that EEG microstates and amplitudes represent complementary brain dynamics at different scales during information processing (Croce et al. 2022). However, Bagdasarov et al. (2024) found a correlation between the locally extracted potential amplitude and globally extracted microstate GEV in the go/no‐go paradigms of image recognition. This inconsistency may be attributed to task specificity. In the present study, the time parameters (duration and frequency) of microstate B were positively correlated with the delta‐, theta‐, and beta‐band powers, whereas the duration of microstate C was negatively correlated with the beta‐band power. In a study exploring cognitive load in creative design tasks, Jia et al. (2021) found that increased power in the delta, theta, and beta bands reflects an increase in cognitive control and suggested that microstate B was positively correlated with cognitive control, which is beneficial for goal orientation. Cognitive control is associated with the periodic activity of large‐scale networks in the beta frequency band, whereas microstate C is negatively correlated with cognitive control (Jia et al. 2021). Cognitive control is a possible explanation for the relationship between microstate parameters and frequency‐band power in the present study, and more comprehensive explanations are worth exploring in future studies.

This study has some limitations. First, we only analyzed and discussed four classes of canonical maps in the microstate analysis. This approach facilitates comparisons across studies (Kim et al. 2021). Therefore, no validation criteria were used to establish the optimal number of maps in the dataset. As a result, the four microstates explained approximately 70% of the variance in the data. Using the optimal number of clusters may provide a better explanation for the variance. However, explaining the cognitive functions of microstates may be challenging when the number of clusters exceeds four (Jia et al. 2021). Thus, the four classes of microstates appear as a trade‐off between explained variance and clarity of cognitive function for mental workload analysis during multitasking. Exploring the optimal number of microstates for changes in mental workload during multitasking warrants further research. Second, we analyzed the changes in frequency‐band power and microstate parameters between the two different mental workloads. Two analysis methods focused on the oscillation of frequency bands and global brain network activation to explore the effects of changes in mental workload during multitasking. However, we did not compare the abilities of the two methods to distinguish between mental workloads. As the basis of the classification research, we conducted a sensitivity analysis. Therefore, classification models and evaluations will be explored in future studies.

## 5 Conclusions

In summary, a high mental workload induced by increasing the number of subtasks during multitasking can manifest as an increase in subjective scores and a decrease in task performance. The EEG frequency‐band power and microstate parameters can detect a high mental workload. In addition, a correlation between EEG frequency‐band power and microstate parameters was detected. These results suggest that power spectral analyses based on frequency‐band oscillations and microstate analyses based on global brain network activation are not completely isolated during multitasking and provide support for mental workload classification based on multiple parameters of different EEG analyses.

## Author Contributions

**Wenbin Li**: methodology; investigation; data curation; formal analysis; writing; original draft preparation; and funding acquisition. **Shan Cheng**: investigation; data curation; formal analysis; writing–original draft preparation. **Jing Dai**: conceptualization; software; resources; visualization; project administration. **Yaoming Chang**: conceptualization; methodology; investigation; formal analysis; writing–review and editing; project administration.

## Ethics Statement

This study complied with the tenets of the Declaration of Helsinki and was approved by the Ethics Committee of the Institutional Review Board of the Air Force Medical University.

## Consent

All participants signed an informed consent form after the study was explained to them and were paid after the experiment, and the rewards were, to some extent, related to task performance.

## Conflicts of Interest

The authors declare no conflicts of interest.

### Peer Review

The peer review history for this article is available at https://publons.com/publon/10.1002/brb3.70216

## Supporting information



Supporting Information

## Data Availability

The data of this study are available from the corresponding author upon a reasonable request.
