# Supplementary material for: Effects of Mental Workload Manipulation on Electroencephalography Spectrum Oscillation and Microstates in Multitasking Environments
Source: Brain Behav. 2025 Jan 8;15(1):e70216. doi: 10.1002/brb3.70216 (PMC11710893; doi:10.1002/brb3.70216)
Supplement: Supplementary file 1 — Supporting Information [file BRB3-15-e70216-s001.docx]

**Supplementary Material**

**Effects of mental workload manipulation on** **electroencephalography** **spectrum** **oscillation and microstates in multitasking environments**

Wenbin Li ^1, #^, Shan Cheng ^2, #^, Jing Dai^3, *^, Yaoming Chang ^1, *^

^1^ Department of Aerospace Hygiene, Faculty of Aerospace Medicine, Air Force Medical University, Xi’an, China

^2^ Department of Aerospace Medical Equipment, Faculty of Aerospace Medicine, Air Force Medical University, Xi’an, China

^3^ Department of Aerospace Ergonomics, Faculty of Aerospace Medicine, Air Force Medical University, Xi’an, China

*** Corresponding author:**

Yaoming Chang

Department of Aerospace Hygiene, Faculty of Aerospace Medicine, Air Force Medical University, No.169, Changle West Road, 710032, Xi’an, China.

Phone: 86-029-84711266

E-mail: changym@fmmu.edu.cn

Jing Dai

Department of Aerospace Ergonomics, Faculty of Aerospace Medicine, Air Force Medical University, No.169, Changle West Road, 710032, Xi’an, China.

Phone: 86-029-84711249

E-mail: daijing@fmmu.edu.cn

^#^ These authors contributed equally to this study and are therefore co-first authors.

**Supplementary Results**

***EEG frequency bands***

Table S1 lists the power of the EEG frequency bands for each electrode. The theta band average power of high mental workload were higher than that of low mental workload in Fp1 (*t* = -3.41, *p-corrected* = 0.004, *Cohen’s d* = 0.24), Fp2 (*t* = -2.84, *p-corrected* = 0.014, *Cohen’s d* = 0.37), Fpz (*t* = -3.70, *p-corrected* = 0.003, *Cohen’s d* = 0.34), F8 (*t* = -2.69, *p-corrected* = 0.019, *Cohen’s d* = 0.32), F3 (*t* = -3.39, *p-corrected* = 0.004, *Cohen’s d* = 0.15), Fz (*t* = -3.43, *p-corrected* = 0.004, *Cohen’s d* = 0.18), FC1 (*t* = -3.31, *p-corrected* = 0.004, *Cohen’s d* = 0.13), T8 (*t* = -4.04, *p-corrected* = 0.001, *Cohen’s d* = 0.34), CP5 (*t* = -2.67, *p-corrected* = 0.019, *Cohen’s d* = 0.14), P7 (*t* = -2.63, *p-corrected* = 0.020, *Cohen’s d* = 0.14), P8 (*t* = -5.17, *p-corrected* < 0.001, *Cohen’s d* = 0.24), P3 (*t* = -3.68, *p-corrected* = 0.003, *Cohen’s d* = 0.13), P4 (*t* = -4.30, *p-corrected* < 0.001, *Cohen’s d* = 0.17), Pz (*t* = -4.03, *p-corrected* = 0.001, *Cohen’s d* = 0.14), POz (*t* = -4.69, *p-corrected* < 0.001, *Cohen’s d* = 0.30), O1 (*t* = -5.05, *p-corrected* < 0.001, *Cohen’s d* = 0.40), O2 (*t* = -4.94, *p-corrected* < 0.001, *Cohen’s d* = 0.35) and Oz (*t* = -6.22, *p-corrected* < 0.001, *Cohen’s d* = 0.48) were higher than that of low mental workload. The alpha band average power of high mental workload in O1 (*t* = -3.24, *p-corrected* = 0.026, *Cohen’s d* = 0.18), O2 (*t* = -3.34, *p-corrected* = 0.026, *Cohen’s d* = 0.10), Oz (*t* = -4.07, *p-corrected =* 0.007, *Cohen’s d* = 0.18) were higher than that of low mental workload. The beta band average power of high mental workload in Fp1 (*t* = -3.95, *p-corrected* = 0.005, *Cohen’s d* = 0.29), Fp2 (*t* = -2.60, *p-corrected* = 0.040, *Cohen’s d* = 0.26), Fpz (*t* = -2.65, *p-corrected* = 0.040, *Cohen’s d* = 0.32), F3 (*t* = -3.09, *p-corrected* = 0.020, *Cohen’s d* = 0.12), Fz (*t* = -4.50, *p-corrected* = 0.002, *Cohen’s d* = 0.21), FC1 (*t* = -3.73, *p-corrected* = 0.007, *Cohen’s d* = 0.17), Cz (*t* = -3.40, *p-corrected* = 0.013, *Cohen’s d* = 0.20), O1 (*t* = -2.81, *p-corrected* = 0.035, *Cohen’s d* = 0.14), O2 (*t* = -2.75, *p-corrected* = 0.035, *Cohen’s d* = 0.10) and Oz (*t* = -3.26, *p-corrected* = 0.015, *Cohen’s d* = 0.16) were higher than that of low mental workload.

***Correlation analysis***

Table S1 lists the correlation coefficients between the frequency band power and microstate parameters. The analysis showed significantly weak correlations between frequency band power and microstate parameters in 27 tests. There was a weak positive correlation between delta band power and the duration of microstate A (*R* = 0.32, *p* = 0.005), transition probability from A to B (*R* = 0.30, *p* = 0.010), and transition probability from B to A (*R* = 0.31, *p* = 0.007). There was a weak negative correlation between the delta band power and the occurrence of microstate C (*R* = -0.37, *p* = 0.001), microstate D (*R* = -0.35, *p* = 0.002), and the transition probability from D to C (*R* = -0.30, *p* = 0.011). There were weak positive correlations between theta band power and the duration of microstate C (*R* = 0.31, *p* = 0.009). There were weak negative correlations between theta band power and the occurrence of microstate A (*R* = -0.35, *p* = 0.002), microstate C (*R* = -0.30, *p* = 0.010), and the transition probability from C to A (*R* = -0.30, *p* = 0.012). There was a weak positive correlation between alpha band power and the duration of microstate B (*R* = 0.38, *p* = 0.001), occurrence of microstate B (*R* = 0.38, *p* = 0.001), coverage of microstate B (*R* = 0.48, *p* < 0.001), transition probability from A to B (*R* = 0.35, *p* = 0.003), and transition probability from B to A (*R* = 0.37, *p* = 0.001). There was a weak negative correlation between alpha band power and the coverage of microstate D (*R* = -0.31, *p* = 0.008), transition probability from A to D (*R* = -0.34, *p* = 0.004), and transition probability from D to A (*R* = -0.35, *p* = 0.003). There was a weak positive correlation between beta band power and the coverage of microstate B (*R* = 0.39, *p* = 0.001), transition probability from A to B (*R* = 0.41, *p* < 0.001), transition probability from B to A (*R* = 0.43, *p* < 0.001), and transition probability from C to A (*R* = 0.34, *p* = 0.003). There was a weak negative correlation between beta band power and the coverage of microstate D (*R* = -0.40, *p* < 0.001), transition probability from A to D (*R* = -0.34, *p* = 0.003), transition probability from C to D (*R* = -0.31, *p* = 0.007), transition probability from D to A (*R* = -0.33, *p* = 0.005), and transition probability from D to C (*R* = -0.31, *p* = 0.009).

**Supplementary Table**

**Table S1. Power of EEG frequency bands in each electrode**

|  | delta band power(uV) | |  | Theta band power(uV) | |  | alpha band power(uV) | |  | beta band power(uV) | |
| --- | --- | --- | --- | --- | --- | --- | --- | --- | --- | --- | --- |
|  | low | high |  | low | high |  | low | high |  | low | high |
| Fp1 | 1.42 ± 0.39 | 1.45 ± 0.42 |  | **0.84 ± 0.26** | **0.91 ± 0.28**** |  | 0.64 ± 0.22 | 0.65 ± 0.20 |  | **0.39 ± 0.11** | **0.43 ± 0.13**** |
| Fp2 | 1.58 ± 0.42 | 1.69 ± 0.58 |  | **0.87 ± 0.22** | **0.87 ± 0.29*** |  | 0.68 ± 0.24 | 0.72 ± 0.22 |  | **0.45 ± 0.16** | **0.49 ± 0.18*** |
| Fpz | 1.40 ± 0.30 | 1.48 ± 0.41 |  | **0.87 ± 0.25** | **0.97 ± 0.32**** |  | 0.64 ± 0.22 | 0.66 ± 0.21 |  | **0.39 ± 0.09** | **0.42 ± 0.11*** |
| F7 | 1.73 ± 0.59 | 1.57 ± 0.42 |  | 0.96 ± 0.37 | 0.90 ± 0.27 |  | 0.80 ± 0.34 | 0.75 ± 0.27 |  | 0.47 ± 0.18 | 0.45 ± 0.15 |
| F8 | 1.64 ± 0.56 | 1.76 ± 0.62 |  | **0.89 ± 0.25** | **0.98 ± 0.30*** |  | 0.75 ± 0.25 | 0.78 ± 0.26 |  | 0.49 ± 0.16 | 0.53 ± 0.17 |
| F3 | 1.33 ± 0.28 | 1.36 ± 0.27 |  | **0.95 ± 0.26** | **0.99 ± 0.29**** |  | 0.65 ± 0.21 | 0.65 ± 0.19 |  | **0.37 ± 0.10** | **0.38 ± 0.09*** |
| F4 | 1.30 ± 0.25 | 1.30 ± 0.26 |  | 0.84 ± 0.22 | 0.86 ± 0.24 |  | 0.62 ± 0.20 | 0.62 ± 0.18 |  | 0.37 ± 0.09 | 0.39 ± 0.11 |
| Fz | 1.40 ± 0.24 | 1.43 ± 0.22 |  | **1.08 ± 0.29** | **1.14 ± 0.34**** |  | 0.69 ± 0.22 | 0.69 ± 0.20 |  | **0.36 ± 0.07** | **0.37 ± 0.07**** |
| FC5 | 1.23 ± 0.37 | 1.23 ± 0.34 |  | 0.72 ± 0.23 | 0.72 ± 0.21 |  | 0.62 ± 0.25 | 0.62 ± 0.22 |  | 0.39 ± 0.16 | 0.39 ± 0.13 |
| FC6 | 1.13 ± 0.29 | 1.20 ± 0.33 |  | 0.66 ± 0.16 | 0.69 ± 0.17 |  | 0.57 ± 0.19 | 0.58 ± 0.19 |  | 0.38 ± 0.11 | 0.40 ± 0.15 |
| FC1 | 1.18 ± 0.22 | 1.20 ± 0.25 |  | **0.85 ± 0.23** | **0.88 ± 0.24**** |  | 0.57 ± 0.18 | 0.58 ± 0.17 |  | **0.28 ± 0.06** | **0.29 ± 0.06**** |
| FC2 | 1.16 ± 0.25 | 1.13 ± 0.20 |  | 0.78 ± 0.21 | 0.78 ± 0.22 |  | 0.55 ± 0.18 | 0.55 ± 0.16 |  | 0.28 ± 0.05 | 0.29 ± 0.05 |
| T7 | 1.47 ± 0.51 | 1.48 ± 0.53 |  | 0.90 ± 0.31 | 0.92 ± 0.34 |  | 0.88 ± 0.44 | 0.90 ± 0.41 |  | 0.64 ± 0.39 | 0.70 ± 0.35 |
| T8 | 1.36 ± 0.37 | 1.47 ± 0.40 |  | **0.83 ± 0.20** | **0.90 ± 0.23**** |  | 0.83 ± 0.42 | 0.86 ± 0.39 |  | 0.62 ± 0.30 | 0.71 ± 0.32 |
| C3 | 1.00 ± 0.24 | 1.02 ± 0.28 |  | 0.62 ± 0.16 | 0.64 ± 0.17 |  | 0.50 ± 0.17 | 0.50 ± 0.16 |  | 0.29 ± 0.07 | 0.29 ± 0.07 |
| C4 | 1.00 ± 0.27 | 0.99 ± 0.26 |  | 0.62 ± 0.19 | 0.61 ± 0.17 |  | 0.50 ± 0.18 | 0.49 ± 0.17 |  | 0.28 ± 0.07 | 0.29 ± 0.08 |
| Cz | 1.12 ± 0.23 | 1.14 ± 0.28 |  | 0.74 ± 0.22 | 0.75 ± 0.22 |  | 0.53 ± 0.17 | 0.53 ± 0.16 |  | **0.24 ± 0.04** | **0.25 ± 0.05*** |
| CP5 | 1.03 ± 0.23 | 1.07 ± 0.24 |  | **0.68 ± 0.18** | **0.71 ± 0.19*** |  | 0.62 ± 0.28 | 0.63 ± 0.26 |  | 0.35 ± 0.12 | 0.36 ± 0.10 |
| CP6 | 1.06 ± 0.28 | 1.08 ± 0.28 |  | 0.72 ± 0.20 | 0.74 ± 0.21 |  | 0.62 ± 0.26 | 0.61 ± 0.23 |  | 0.36 ± 0.12 | 0.37 ± 0.10 |
| CP1 | 0.99 ± 0.22 | 1.00 ± 0.29 |  | 0.60 ± 0.16 | 0.62 ± 0.18 |  | 0.48 ± 0.18 | 0.48 ± 0.18 |  | 0.24 ± 0.05 | 0.24 ± 0.06 |
| CP2 | 1.02 ± 0.27 | 1.00 ± 0.26 |  | 0.61 ± 0.17 | 0.61 ± 0.17 |  | 0.50 ± 0.18 | 0.49 ± 0.17 |  | 0.23 ± 0.05 | 0.24 ± 0.05 |
| P7 | 1.62 ± 0.36 | 1.61 ± 0.33 |  | **1.05 ± 0.25** | **1.09 ± 0.27*** |  | 0.97 ± 0.50 | 0.96 ± 0.42 |  | 0.58 ± 0.29 | 0.59 ± 0.22 |
| P8 | 1.57 ± 0.38 | 1.60 ± 0.34 |  | **1.06 ± 0.27** | **1.13 ± 0.30***** |  | 0.92 ± 0.35 | 0.93 ± 0.29 |  | 0.57 ± 0.18 | 0.59 ± 0.16 |
| P3 | 1.18 ± 0.34 | 1.21 ± 0.33 |  | **0.75 ± 0.23** | **0.78 ± 0.24**** |  | 0.65 ± 0.25 | 0.64 ± 0.23 |  | 0.34 ± 0.10 | 0.35 ± 0.10 |
| P4 | 1.30 ± 0.39 | 1.30 ± 0.37 |  | **0.81 ± 0.24** | **0.86 ± 0.24***** |  | 0.72 ± 0.28 | 0.70 ± 0.24 |  | 0.37 ± 0.14 | 0.34 ± 0.12 |
| Pz | 1.23 ± 0.30 | 1.27 ± 0.30 |  | **0.72 ± 0.21** | **0.75 ± 0.21**** |  | 0.62 ± 0.21 | 0.61 ± 0.19 |  | 0.29 ± 0.08 | 0.29 ± 0.07 |
| POz | 1.41 ± 0.28 | 1.48 ± 0.36 |  | **0.90 ± 0.22** | **0.97 ± 0.24***** |  | 0.82 ± 0.27 | 0.84 ± 0.26 |  | 0.48 ± 0.20 | 0.49 ± 0.21 |
| O1 | 1.61 ± 0.24 | 1.68 ± 0.25 |  | **1.11 ± 0.21** | **1.20 ± 0.21***** |  | **1.15 ± 0.35** | **1.21 ± 0.33*** |  | **0.84 ± 0.31** | **0.88 ± 0.31*** |
| O2 | 1.61 ± 0.38 | 1.66 ± 0.33 |  | **1.08 ± 0.24** | **1.17 ± 0.25***** |  | **1.11 ± 0.55** | **1.16± 0.56*** |  | **0.77 ± 0.40** | **0.80 ± 0.41*** |
| Oz | 1.56 ± 0.24 | 1.64 ± 0.26 |  | **1.06 ± 0.21** | **1.16 ± 0.21***** |  | **1.07 ± 0.37** | **1.14 ± 0.37**** |  | **0.75 ± 0.26** | **0.79 ± 0.26*** |

**p* < 0.05, ***p* < 0.01, ****p* < 0.001, as compared with low mental workload, FDR corrected. Statistically significant differences are indicated in boldface.

**Table S2. Correlation coefficient between frequency bands power and microstates parameters**

| Parameters | Delta band | Theta band | Alpha band | Beta band |
| --- | --- | --- | --- | --- |
| Duration of microstate A | **0.32**** | 0.09 | -0.11 | -0.17 |
| Duration of microstate B | **0.51***** | **0.54***** | **0.38**** | 0.03 |
| Duration of microstate C | 0.18 | **0.31**** | -0.24 | **-0.51***** |
| Duration of microstate D | -0.01 | 0.04 | -0.12 | -0.20 |
| Occurrence of microstate A | -0.18 | **-0.35**** | -0.06 | 0.26* |
| Occurrence of microstate B | -0.13 | -0.06 | **0.38**** | **0.52***** |
| Occurrence of microstate C | **-0.37**** | **-0.30**** | -0.08 | 0.11 |
| Occurrence of microstate D | **-0.35**** | -0.22 | -0.26* | -0.14 |
| Coverage of microstate A | 0.12 | -0.15 | -0.11 | 0.02 |
| Coverage of microstate B | 0.27* | 0.23 | **0.48***** | **0.39*** |
| Coverage of microstate C | -0.28* | -0.18 | -0.16 | -0.09 |
| Coverage of microstate D | -0.18 | 0.01 | **-0.31**** | **-0.40***** |
| Transition probability from A to B | **0.30*** | 0.13 | **0.35**** | **0.41***** |
| Transition probability from A to C | -0.15 | -0.27* | -0.21 | -0.03 |
| Transition probability from A to D | 0.01 | -0.1 | **-0.34**** | **-0.34**** |
| Transition probability from B to A | **0.31**** | 0.13 | **0.37**** | **0.43***** |
| Transition probability from B to C | -0.09 | -0.01 | 0.24* | 0.28* |
| Transition probability from B to D | -0.02 | 0.20 | 0.10 | -0.08 |
| Transition probability from C to A | -0.18 | **-0.30*** | -0.26* | -0.09 |
| Transition probability from C to B | -0.09 | 0.01 | 0.29* | **0.34**** |
| Transition probability from C to D | -0.29* | -0.14 | -0.29* | **-0.31**** |
| Transition probability from D to A | 0.01 | -0.09 | **-0.35**** | **-0.33**** |
| Transition probability from D to B | -0.02 | 0.19 | 0.10 | -0.10 |
| Transition probability from D to C | **-0.30*** | -0.14 | -0.29* | **-0.31**** |

**p* < 0.05, ***p* < 0.01, ****p* < 0.001. Significant moderate correlations are shown in bold and grey. Weak correlations are indicated in boldface.
